# IGF2 reduces meiotic defects in oocytes from obese mice and improves embryonic developmental competency

**DOI:** 10.1186/s12958-022-00972-9

**Published:** 2022-07-14

**Authors:** Yanling Wan, Tahir Muhammad, Tao Huang, Yue Lv, Qianqian Sha, Shuang Yang, Gang Lu, Wai-yee Chan, Jinlong Ma, Hongbin Liu

**Affiliations:** 1 Center for Reproductive Medicine, Shandong University, Jinan, Shandong China; 2 Key Laboratory of Reproductive Endocrinology of Ministry of Education, Shandong University, Jinan, Shandong China; 3 Shandong Key Laboratory of Reproductive Medicine, Jinan, Shandong China; 4 Shandong Provincial Clinical Research Center for Reproductive Health, Jinan, Shandong China; 5 Shandong Technology Innovation Center for Reproductive Health, Jinan, Shandong China; 6 National Research Center for Assisted Reproductive Technology and Reproductive Genetics, Shandong University, Jinan, Shandong China; 7 Research Unit of Gametogenesis and Health of ART-Offspring, Chinese Academy of Medical Sciences, Beijing, China; 8 Fertility Preservation Laboratory, Reproductive Medicine Center, Guangdong Second Provincial General Hospital, Guangzhou, Guangdong China; 9 Department of Physiology School of Basic Medical Sciences Cheeloo College of Medicine Shandong University, Jinan, Shandong China; 10 CUHK-SDU Joint Laboratory on Reproductive Genetics, School of Biomedical Sciences, Faculty of Medicine, The Chinese University of Hong Kong, Hong Kong, China; 11 Institute of Molecular Biology and Biotechnology, The University of Lahore, Lahore, Pakistan

**Keywords:** Maternal obesity, Oocyte quality, IGF2, Meiotic defects

## Abstract

**Background:**

Maternal obesity is a global issue that has devastating effects across the reproductive spectrum such as meiotic defects in oocytes, consequently worsening pregnancy outcomes. Different studies have shown that such types of meiotic defects originated from the oocytes of obese mothers. Thus, there is an urgent need to develop strategies to reduce the incidence of obesity-related oocyte defects that adversely affect pregnancy outcomes. Multiple growth factors have been identified as directly associated with female reproduction; however, the impact of various growth factors on female fertility in response to obesity remains poorly understood.

**Methods:**

The immature GV-stage oocytes from HFD female mice were collected and cultured in vitro in two different groups (HFD oocytes with and without 50 nM IGF2), however; the oocytes from ND mice were used as a positive control. HFD oocytes treated with or without IGF2 were further used to observe the meiotic structure using different analysis including, the spindle and chromosomal analysis, reactive oxygen species levels, mitochondrial functional activities, and early apoptotic index using immunofluorescence. Additionally, the embryonic developmental competency and embryos quality of IGF2-treated zygotes were also determined.

**Results:**

In our findings, we observed significantly reduced contents of insulin-like growth factor 2 (IGF2) in the serum and oocytes of obese mice. Our data indicated supplementation of IGF2 in a culture medium improves the blastocyst formation: from 46% in the HFD group to 61% in the HFD + IGF2-treatment group (50 nM IGF2). Moreover, adding IGF2 to the culture medium reduces the reactive oxygen species index and alleviates the frequency of spindle/chromosome defects. We found increased mitochondrial functional activity in oocytes from obese mice after treating the oocytes with IGF2: observed elevated level of adenosine triphosphate, increased mitochondrial distribution, higher mitochondrial membrane potentials, and reduced mitochondrial ultrastructure defects. Furthermore, IGF2 administration also increases the overall protein synthesis and decreases the apoptotic index in oocytes from obese mice.

**Conclusions:**

Collectively, our findings are strongly in favor of adding IGF2 in culture medium to overcome obesity-related meiotic structural-developmental defects by helping ameliorate the known sub-optimal culturing conditions that are currently standard with assisted reproduction technologies.

**Supplementary Information:**

The online version contains supplementary material available at 10.1186/s12958-022-00972-9.

## Introduction

Oocyte quality is a major indicator to predict the early embryonic developmental competency and pregnancy outcomes that collectively impacts the overall female fertility. Several factors are associated with oocyte quality, however, obesity is considered a major deleterious factor and a worldwide major public health concern that is directly linked to declining oocyte quality [1, 2]. Maternal obesity is highly accompanied by reduced female reproductive outcomes such as congenital defects, early pregnancy losses, and developing neonatal conditions [3, 4]. Different studies have shown that defects in female fertility associated with obesity probably originated from the oocytes of the obese mother [5, 6]. Notably, it was observed that higher pregnancy failure rate might be reversed in part if good quality donor oocytes were used rather than using autologous oocytes during assisted reproductive technology (ART) [7].

Several meiotic defects were noted in oocytes from obese mothers including impaired spindle assembly, chromosome misalignment, and mitochondrial dysfunction [1, 8, 9]. Mitochondria, a major source of energy in oocytes for different metabolic and cellular activities are required for regulating cellular calcium homeostasis, apoptotic level, redox homeostasis, and cellular translation during oocytes and embryo development [10–12]. It was shown that mitochondrial dysfunction in oocytes from obese women is associated with several meiotic defects including, higher spindle and chromosomal abnormalities, increased reactive oxygen species (ROS) index, and ultimately impaired regulation of molecular and cellular processes that consequently cause female infertility [13, 14]. Such type of meiotic defects in oocytes from obese women promotes the chances of miscarriage, infertility, and congenital malformation. Altogether, there is an urgent need to develop strategies to reduce the incidence of obesity-related oocyte defects that adversely affect pregnancy outcomes. Therefore, understanding the obesity-related impact on oocyte quality would very likely have a significant impact on ART outcomes.

Growth factors are naturally occurring substances that are involved in cell proliferation and cell differentiation by interacting with its specific receptors. Among all reproduction-related known growth factors belonging to the insulin-like growth factors (IGFs) family, insulin-like growth factor 2 (IGF2) is specifically well-known regarding female fertility; its expressions are prominent in germ cells including, granulosa cells, follicles, oocytes, and embryos of different mammalian species [15–18], that critically triggers the human ovarian system [16]. IGF2 has played an essential role in different reproductive phenomena including follicular growth, oocytes, and embryo development, increasing fetal growth with reduced placental apoptosis [19–22]. Multitude studies in animal and human models have provided evidence about the potential impact of IGF2 on oocytes and embryos developmental competency [19, 23, 24]. Furthermore, recent reports also have shown that IGF2 enhances the mitochondrial functional activity in adult neuronal culture-derived cell lines: by increasing mitochondrial membrane potential (MMPs), mitochondrial distribution, and also reducing oxidative stress index [25, 26]. Despite these several basic attributes and knowledge, the application potential of IGF2 in female fertility is still needed to be investigated, so rare is known how IGF2 put a cellular and molecular impact on the developmental competency of oocytes from obese mothers and may confer any effect on oocytes meiotic structure that ultimately affect the overall implantation success and pregnancy outcomes.

After detecting significantly reduced concentrations of IGF2 in serum and oocytes samples from obese mice, we found increased blastocyst formation and reduced abnormalities in spindle and chromosome organization of oocytes from obese mice with the supplementation of IGF2 to the culture medium. Besides, we found that IGF2 supplementation improves the overall mitochondrial function, by for example increasing ATP levels, elevating immunoreactivity of mitochondria in oocytes, increasing MMPs, and increasing overall protein synthesis. Collectively, our findings are strongly in favor of adding IGF2 in culture medium to overcome obesity-related meiotic structural-developmental defects by helping ameliorate the known sub-optimal culturing conditions that are currently standard with ART.

## Materials and methods

### Mice

ICR female mice of age 3 weeks were purchased from Charles River Laboratories, China Inc., and were housed in the animal room by providing 12-h light: 12-h dark cycle at constant temperature (22 °C) and controlled humidity. These females were randomly divided into two different groups; the first group received a standard normal diet of rodents (D1415, Beijing HFK Bioscience Co. Ltd) and named as ND group, while the second group was provided with a high-fat diet (D12492, Research Diets, Inc. with 60% kcal from fat) and was considered as HFD group for up to 16 weeks.

### Measurement of blood glucose level by glucose tolerance test

For the measurement of glucose by using glucose tolerance tests (GTT), glucose (2 g/kg body weight) was injected into the mice intraperitoneally after 12 h of fasting. Blood was collected after puncturing the tail vein at the different time frames and the concentration of glucose was detected by the Hemocue B glucose analyzer.

### Oocytes collection and maturation

To collect the GV-stage oocytes, ND and HFD female mice were superstimulated with 5 IU pregnant mare’s serum gonadotropin (PMSG) injection [27]. Female mice were sacrificed after 48 h of PMSG injection and GV-stage oocytes were collected and washed thoroughly to remove cumulus cells by genital pipetting and then randomly oocytes are divided into two groups. Oocytes retrieved from HFD mice were cultured in a small drop of M16 (M7292; Sigma-Aldrich) with or without *50 nM IGF2 (100–12, Peprotech) by maintaining* 5% CO_2_ at 37 °C. For MII-stage oocytes collection, 5 IU human chorionic gonadotrophin (hCG) was injected after 44 h of PMSG and then oocytes were collected after 16 h of hCG injection. These oocytes were used for further in vitro fertilization (IVF) related experiments.

### Culturing of zygotes

*For the collection of MII-stage oocytes, mice were superstimulated* with 5 IU PMSG followed by 5 IU human chorionic gonadotrophin (hCG) after 44 h. MII-stage oocytes were collected *from ND and HFD female mice* after 16 h of hCG and used for in vitro fertilization (IVF) experiment. Oocytes from both groups were *mixed with the sperms from wild-type (WT) males to perform an IVF experiment. After fertilization, the zygotes obtained from HFD mice were randomly allocated into two groups and* were cultured in a small drop of M16 medium with or without 50 nM IGF2 and maintained temperature up to *37* °C *in 5% CO*_*2,*_ *5% O*_*2*_ *to observe their embryonic developmental potential.* Morphology and developmental competency of embryos were observed by using a *stereomicroscope (Nikon SMZ1500).*

### Measurement of IGF2 concentration from serum sample

Serum IGF2 concentration was measured by following the protocol using an ELISA kit (RnD system, MG200). Briefly, blood was obtained from ND and HFD mice and kept at room temperature for 1 h. These blood samples were further centrifuged at 3000×g for 10 min at 4 °*C. ELISA* assay was performed using serum sample and IGF2 concentration was measured in triplicate. The IGF2 contents were measured by using a formula after generating a standard curve.

### RNA extraction and qRT-PCR validation

REPLI-g WTA Single Cell Kit (Qiagen) was used for extraction of total RNA and acquisition of ultimately cDNA in accordance with the protocol by the manufacturer. *Power SYBR Green Master Mix dye (Takara) was used to observe the qRT-PCR analysis by using Roche 480 PCR system*. To measure the values of genes specific primers, the values of qRT-PCR reactions were measured in triplicate. The value of mRNA was calculated by normalizing the actin (internal control) level of endogenous mRNA using Microsoft Excel. The sequence of all the indicated primers were listed (Table S1, Supporting Information)*.*

### Immunofluorescence

For the determination of specific relevant protein, oocytes from ND, HFD, and IGF2-treated group were fixed in 4% paraformaldehyde (PFA) for 30 min at room temperature and then permeabilized with 0.3% Triton X-100 for 20 min. These oocytes were washed by using washing buffer and then were blocked in PBS containing 1% BSA. To observe the spindle organization, oocytes were washed and cultured in a medium containing fluorescein isothiocyanate (FITC)-conjugated anti-mouse Alpha tubulin (1:200 dilution, Sigma) antibody. After several washing, these oocytes were cultured in secondary antibodies. DAPI (Sigma) or Hoechst (Sigma) staining is used for 10 min at room temperature to determine the DNA structure and chromosomal alignment. Oocytes were shifted on the glass slides by using a mini drop of anti-fade medium (Vectashield, CA, USA) and a confocal laser microscope (Andor Technology Ltd., Belfast, UK) was used to examine the structure.

For blastocysts, the blastocysts were fixed with 4% PFA overnight at 4 °C and then permeabilized for 40 min. These blastocysts were washed and blocked in PBS containing 1% BSA, then incubated with CDX2 (1:500 dilution, Abcam) for 1 h. After several times washing, the blastocysts were incubated with secondary antibodies. The nuclei were stained with DAPI, then mounted on the glass slides. The blastocysts were observed with a confocal laser microscope (Andor Technology Ltd., Belfast, UK).

### Determination of ATP levels

The level of ATP in IGF2-treated HFD oocytes at the MII-stage was determined by using an ATP testing assay kit (Beyotime). Briefly, oocytes were incubated in lysis buffer and then used centrifugation 12,000×g for 10 min. The supernatant was collected and then mixed with a testing buffer. The total ATP value was examined with the help of a luminescence detector (EnSpire Multimode Plate Reader). A standard curve with the range of 0.01 mM to 1 m was used to determine the total ATP contents.

### ROS evaluation

To assess the level of ROS production, HFD oocytes treated with and without IGF2 were used and ROS index was measured by ROS assay kit (Beyotime) following the manufacturer’s protocols. Briefly, oocytes at MII-stage from HFD mice treated with and without IGF2 were cultured in a medium supplemented with 10 μM, 2′,7′ dichlorofluorescein diacetate (DCFH-DA) at 37 °C in 5% CO_2_ for 30 minutes. The oocytes were transferred on glass slides, following three washings, and images were taken under a confocal laser microscope (Andor Technology Ltd., Belfast, UK).

### Assessment of mitochondrial distribution and mitochondrial membrane potential

To evaluate the mitochondrial distribution, MII-stage oocytes of ND and HFD oocytes treated with and without IGF2 were incubated with 400 nmol/L Mitotracker Green FM (Invitrogen) diluted in PBS for 30 minutes at 37 °C. The oocytes were treated with 10 μM JC-1 (Beyotime Institute of Biotechnology) mixed with M16 culture medium and incubated at 37 °C for 30 min for the determination of mitochondrial membrane potential. Oocytes were shifted onto the glass slides and examined immediately (Andor Technology Ltd., Belfast, UK) after three washing with PBS. The ratio of red and green fluorescents intensities were used as an indicator of mitochondrial membrane potential in the oocytes sample.

### Annexin-V analysis

To detect the early-apoptosis index, MII-stage oocytes from ND, HFD, and IGF2-treated groups were assessed by an Annexin V assay kit (Beyotime) following the manufacturer’s instructions. Briefly, the MII-stage oocytes were treated with 195 μl binding buffer containing 5 μl of Annexin V-mCherry in the dark for 15 minutes at room temperature. The zona pellucida of oocytes were removed by Tyrode’s Solution (Sigma) and shifted to a M16 drop in living cell culture dishes. The Annexin-V fluorescent was examined immediately with a confocal system (Andor Technology Ltd., Belfast, UK).

### Detection of protein synthesis

To examine the total protein synthesis in ND, HFD, and IGF2-treated HFD oocytes, the Click-iT protein synthesis assay kit (C10428, Life Technologies) was used following the manufacturer’s instructions as described previously [28]. Briefly, the MII-stage oocytes from different groups were exposed to 50 μM HPG for the period of 1 hr. at 37 °C with 5% CO2. After treatment, 3.7% paraformaldehyde was used for oocytes fixation and permeabilization was performed with Triton X-100 at room temperature for 20 min. HPG signal intensity is indicative of the total protein synthesis index in oocytes.

### Transmission Electron microscope evaluation

To examine the mitochondrial ultrastructure of ND and HFD oocytes treated with and without IGF2, transmission electron microscopy (TEM) was performed as described previously [29]. Briefly, MII-stage oocytes were fixed in fixation solution and a KGN cell line was used to cover the oocytes. The samples were processed and visualized and images were captured with a transmission electron microscope (TEM, JEOL). The percentages of abnormal mitochondria of oocytes belonging to different groups were counted from electron micrographs at 5000-fold magnification.

### Statistical analysis

Data were evaluated for statistical analysis by using mean ± SEM of three independent experiments/samples unless otherwise specified. Two-tailed unpaired Student’s t-tests or one-way ANOVA statistical tool was applied to determine the group comparisons value when necessary. In all cases, the difference was considered statistically at *p* < 0.05. All analyses were performed using the GraphPad Prism 9.0.0 (GraphPad Software, San Diego, CA, USA).

## Results and discussion

### Establishment of obese mice model induced by a high-fat diet

To develop a mice model of obesity, a high-fat diet was fed to ICR female mice (“HFD mice”) for 12 weeks starting from the age of 4 weeks. Control mice were established by feeding a normal diet (“ND mice”). As expected, the female HFD mice became obese, with a significantly higher average body weight than the ND mice (Fig. S1A, B, C), and the HFD mice displayed both glucose intolerance and insulin resistance at multiple different time points (Fig. S1D, E, and F). These HFD and ND mice were used for the following experiments.

### Reduced IGF2 contents in serum and oocytes sample of obese mice

**Fig. 1:**
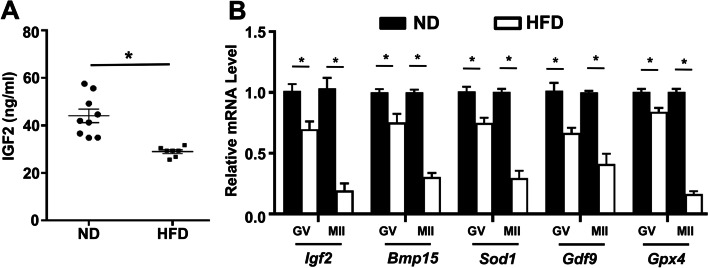
Oocytes from obese mice display reduced serum IGF2 levels and reduced *IGF2* mRNA expressions **(A)** Serum IGF2 contents in mice with normal diet (ND) and high-fat diet (HFD), measured by ELISA. *n* = 9 female mice for each group. **B** qPCR data indicating the mRNA expressions of *IGF2* and target genes in mouse oocytes at different stages from ND and HFD mice. *p < 0.05. Student’s *t-*test (two-tailed) was used for statistical analysis. Error bars indicate the SEM

Given reports from previous studies about the involvement of IGF2 in promoting female fertility, we examined the contribution of this growth factor in the development of oocytes in obese mice. First, we used ELISA to measure the serum IGF2 level from ND and HFD and found a significant reduction in the serum IGF2 concentration in HFD mice (Fig. 1A). Previous studies have reported reduced serum IGF2 levels in women with increased body weight [30, 31]. Another study reported that patients with metabolic syndrome display reduced serum IGF2 levels [32]. Further supporting an obesity-related decline in IGF2 levels, a qPCR analysis showed that *Igf2* mRNA expression was significantly reduced in oocytes (GV-stage and MII-stage oocytes) collected from HFD mice (Fig. 1B). In addition, we found a significantly reduced level of known antioxidant genes previously implicated in IGF2-mediated impacts on oocytes, including *Bmp15, Sod1, Gdf9*, and *Gpx4* (Fig. 1B). Our initial screening based on in vivo data from ND and HFD mice is consistent with these findings, showing a reduced level of IGF2 in serum with relatively decreased *Igf2* mRNA expression in oocytes retrieved from obese mice. Altogether, these findings suggest that a decreased level of IGF2 contents may be the possible cause of impairment in oocyte development of obese mice.

### IGF2 administration improves the embryonic developmental potential of obese mice

**Fig. 2:**
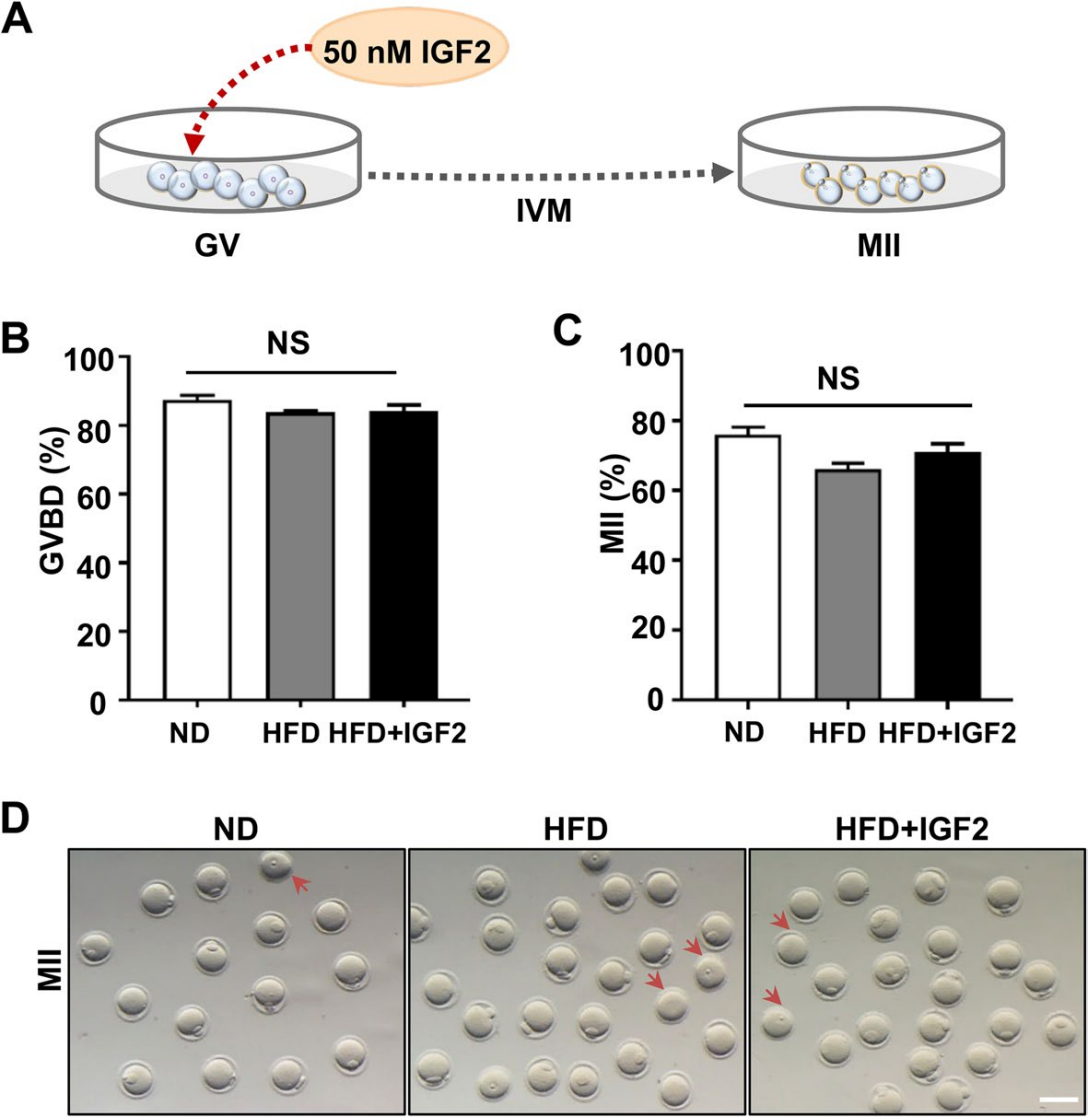
Role of IGF2 in oocytes maturation of obese mice **(A)** Schematic diagram indicating treatment plan of oocytes in culture medium in vitro. **B**, **C** Percentage of GVBD and MII-stage oocytes from ND (*n* = 79) or HFD (*n* = 150) mice, and of oocytes from HFD mice given 50 nM IGF2 to the culture medium (“HFD + IGF2”). **D** Morphology of oocytes cultured in vitro and examined at the MII developmental stage. The arrows point to oocytes that are unable to transform into MII-stage. Scale bar, 100 μm. NS, not significant. One-way ANOVA test was used for statistical analysis. Error bars indicate the SEM

Previous reports have shown the ability of IGF2 to increase the meiotic maturation and early embryonic development of mice and porcine oocytes when added to the culture medium [21, 33]. To examine the IGF2 impact on oocytes development, oocytes at GV-stage were retrieved from HFD mice and cultured in a medium containing 50 nM IGF2 and without IGF2 (Fig. 2A). We found that adding IGF2 to the culture medium had no effect on oocytes maturation: no significant differences (*p* > 0.05) in the percentage of germinal vesicle breakdown (GVBD) or the polar body (Pb1) extrusion rate were noticed after 16 h of in vitro culture (Fig. 2B, C, and D).

**Fig. 3:**
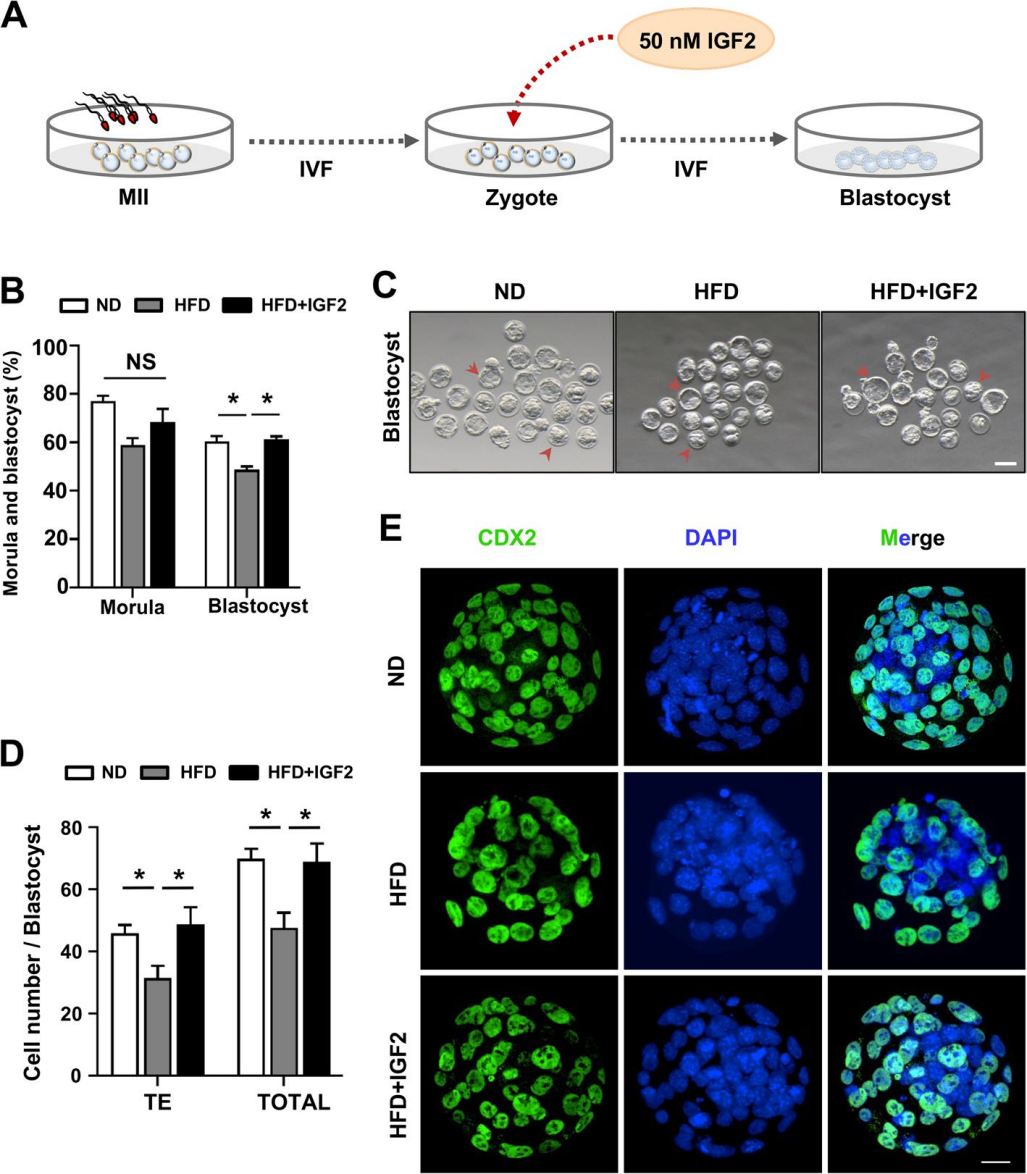
IGF2 administration improves the embryonic developmental competency of oocytes from obese mice **(A)** Schematic diagram indicating treatment plan of embryos in culture medium in vitro. **B** Quantitative analysis of morula and blastocyst in ND (*n* = 72), HFD (*n* = 57), and HFD+ IGF2 (*n* = 49). **C** Morphology of embryos cultured in vitro and examined at the blastocyst developmental stage. The arrows point to embryos that developed into blastocysts; the arrowheads showing the embryos which failed to transform into blastocysts. Scale bar, 300 μm. **D** Quantification of the total and TE number of cells in ND (*n* = 37), HFD (*n* = 20) and HFD + IGF2 (*n* = 26) blastocysts. **E** Representative images of blastocysts of the ND, HFD, and HFD + IGF2 groups. The trophectoderm (TE) was stained with CDX2 (green), and chromosomes were counterstained with DAPI (blue). Scale bar, 20 μm. NS, not significant. *p < 0.05. One-way ANOVA test was used for statistical analysis. Error bars indicate the SEM

We further investigated whether IGF2 administration could improve the embryonic developmental potential of oocytes from HFD mice. To do this, mice were superstimulated and MII-stage oocytes *from ND and HFD female mice were collected and mixed with the sperms from WT males to perform an IVF experiment. After fertilization, the* zygote-stage embryos were cultured in M16 medium with or without 50 nM IGF2 (Fig. 3A) and zygotes from ND mice were used as control. We found that adding IGF2 in the culture medium significantly (*p* < 0.05) increased the percentage of the blastocyst (from 49 to 61% in the IGF2 group) (Fig. 3B and C) (Table S2, Supporting Information). Notably, the untreated HFD zygotes were mostly arrested at the compact morula-stage (Fig. 3C). Note that the 50 nM IGF2 concentration was selected based on previous reports [19, 33]. It is to be noted that IGF2-treatment was performed at two different time points (GV-stage and zygote-stage) as MII-stage oocytes obtained from IVM were not be proceeded for an IVF experiment due to the limited number of immature oocytes retrieved from obese mice with their compromised quality and that may be the limiting part of our study. A role for IGF2 in improving blastocyst formation and quality has been demonstrated in murine and human experiments [19, 24, 34]. Clinically, the level of IGF2 in follicular fluid of human females has been proved as an indicator to judge the growth potential of human oocytes, indicating IGF2 may be considered a valuable biomarker for oocytes development [23]. Previous studies have shown that the morphological parameters of blastocysts such as inner cell mass (ICM) and trophectoderm (TEs) are informative predictors to assess the clinical pregnancy and live birth outcomes in blastocyst transfer cycles [35, 36]. We, therefore, assessed the quality of embryos treated with IGF2 by examining the TEs and total cell number per blastocyst using CDX2 staining with DAPI counterstaining (Fig. 3E). Adding IGF2 in the culture medium improved blastocyst quality, significantly increasing the total number of cells and TEs per blastocyst (Fig. 3D) (Table S2, Supporting Information). Thus, our results suggest that IGF2 may increase embryonic developmental competency and may also improve the quality of embryos from obese mice.

### IGF2 ameliorates spindle and chromosome defects while also reducing oxidative stress levels in oocytes from obese mice

**Fig. 4:**
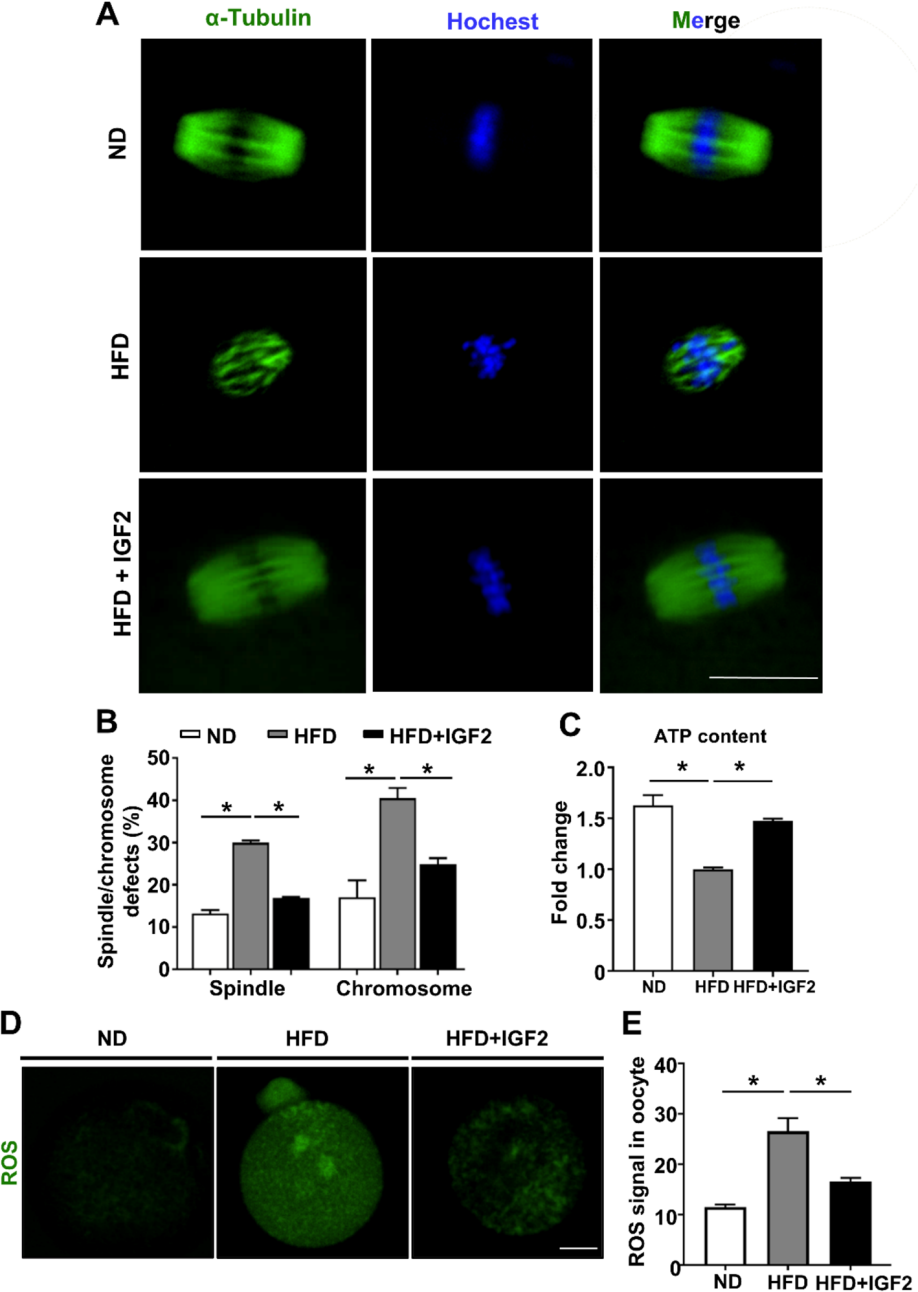
IGF2 ameliorates the quality of obese mice oocytes by reducing oxidative stress. **A** Morphology of spindle and chromosome organization in ND, HFD, and HFD + IGF2 oocytes. *α*-tubulin antibody (green) was used to stained the spindles while chromosomes were counterstained with Hoechst 33342 (blue). Scale bar = 10 μm. **B** Quantification of oocytes with spindle/chromosomes defects in ND (*n* = 38), HFD (*n* = 76), and HFD + IGF2 (*n* = 72) oocytes. **C** Quantification of ROS (DCFH-DA staining) signals in ND (*n* = 10), HFD, (n = 10), and HFD + IGF2 (n = 10) oocytes. **D** Representative confocal images showing CM-H2DCFDA fluorescence (green) in ND, HFD, and HFD + IGF2 oocytes. Scale bar = 20 μm. **E** Adenosine triphosphate (ATP) content in ND (*n* = 50), HFD (n = 50), and HFD + IGF2 (n = 50) oocytes. *p < 0.05. One-way ANOVA test was used for statistical analysis. Error bars indicate the SEM

Previously, it was shown that several variables such as spindle and chromosome morphology and mitochondrial potential activity are informative predictors to evaluate the quality of the oocyte, and reports about obese mouse oocytes have shown spindle and chromosomal defects at the higher index that are accompanied by an increased extent of oxidative stress [1, 37]. We explored the impact of IGF2 supplementation to the culture medium on the quality of oocytes from HFD mice from GV to MII-stage. To assess the spindle morphology and chromosomal alignment, MII-stage oocytes were stained with anti-α-tubulin antibody and were counterstained with Hoechst. Confocal microscopy coupled with quantitative analysis showed a reduced extent of spindle and chromosome alignment abnormalities in the HFD + IGF2 oocytes compared to the untreated HFD oocytes (Fig. 4A and B). Unlike the HFD oocytes, the HFD + IGF2 exhibited barrel-shaped spindles with well-aligned chromosomes that appeared similar to the structures of the ND oocytes (Fig. 4A).

In addition, we observed that HFD + IGF2 oocytes had significantly increased (*p* < 0.05) ATP levels compared to HFD oocytes (Fig. 4C). Given the involvement and known beneficial effect of IGF2 in redox homeostasis in aged mice oocytes [38], we next examined whether IGF2 administration could reduce the oxidative level of HFD oocytes. DCFH-DA staining showed a significantly reduced (*p* < 0.05) ROS level in HFD + IGF2 oocytes compared to HFD oocytes (Fig. 4D and E). Altogether, these findings support the functional activity of IGF2 in improving embryonic developmental competency of oocytes by reducing ROS index that is accompanied by lesser spindle assembly and chromosome alignment defects.

### IGF2 improves mitochondrial function and protein translation in oocytes from obese mice

**Fig. 5:**
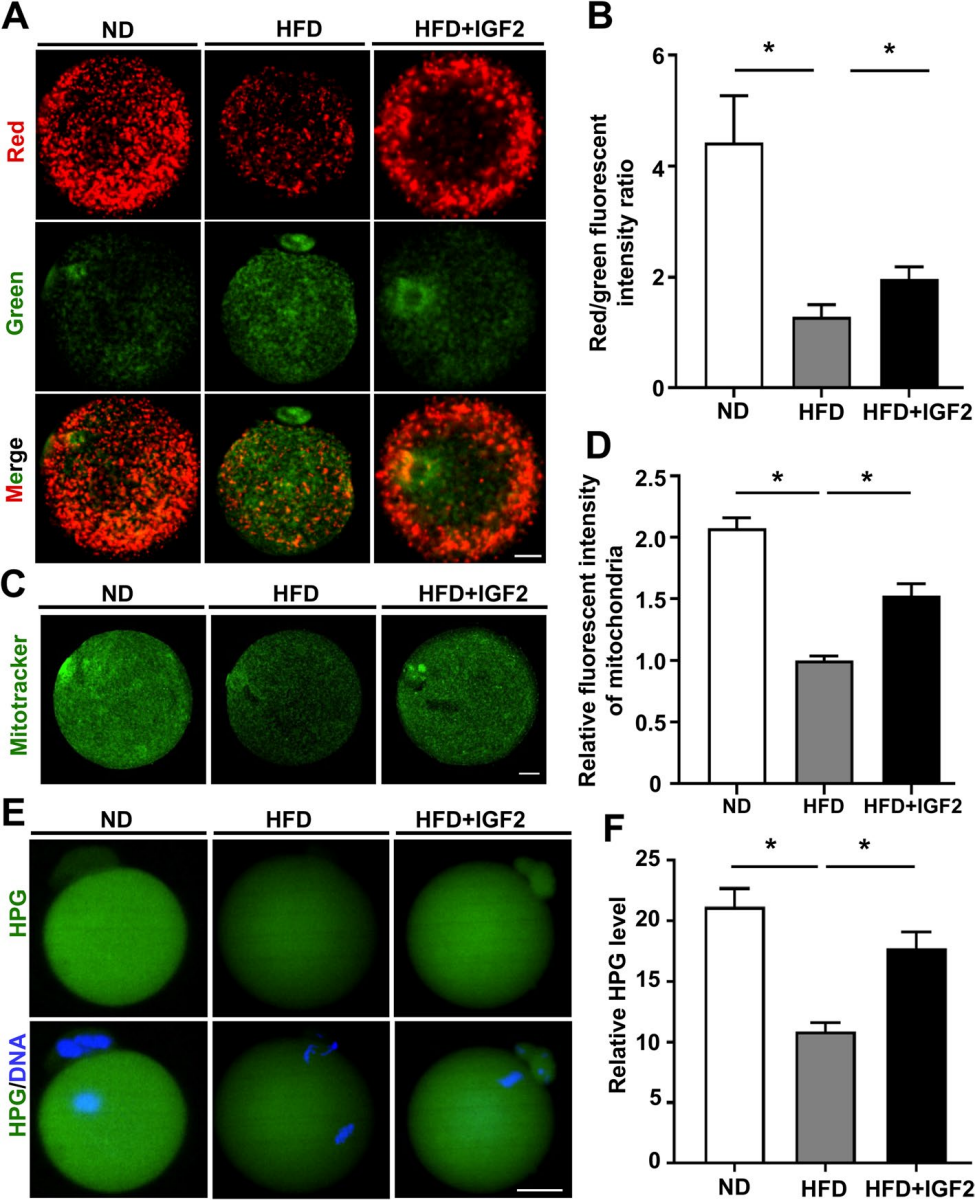
IGF2 increases mitochondrial activity in oocytes from obese mice (**A**) ND, HFD, and HFD + IGF2 oocytes were stained with JC-1 to test the mitochondrial membrane potential (MMP). Scale bar = 10 μm. **B** Quantification of the ratio of red to green fluorescence intensity in ND (*n* = 20), HFD (*n* = 22), and HFD + IGF2 (*n* = 20) oocytes. **C** Mitochondria were labeled with mitotracker Green FM (green). Scale bar = 20 μm. **D** Quantification of mitochondrial distribution signals in ND (*n* = 148), HFD (*n* = 54), and HFD + IGF2 (*n* = 56) oocytes. **E** Immunofluorescence of HPG (green) showing the overall translation levels in MII-stage ND, HFD, and HFD + IGF2 oocytes. Chromosomes were counterstained with DAPI (blue). Scale bar = 20 μm. **F** HPG signal intensity quantification in ND (*n* = 16), HFD, (*n* = 20) and HFD + IGF2 (*n* = 17) oocytes. *p < 0.05. One-way ANOVA test was used for statistical analysis. Error bars indicate the SEM

Mitochondrial function is a known predictor of oocytes quality [39] and previous studies of oocytes from obese mice have reported extensive mitochondrial functional defects including reduced MMPs and reduced mitochondrial distribution [13, 14]. Staining with common dyes for monitoring mitochondrial dynamics (Mitotracker and JC-1) showed that HFD + IGF2 oocytes had significantly higher MMPs compared to HFD oocytes and indicated that the mitochondrial distribution of the HFD + IGF2 oocytes resembled the ND oocytes more closely than the HFD oocytes (Fig. 5A-D). A study based on adult neuronal cells indicated the functional impact of IGF2 in the culture medium to promote mitochondrial functional activity by increasing the MMPs level and also decreasing oxidative damage [25]. Mitochondrial functional activity is essential for protein homeostasis and disrupted protein metabolism has been reported for oocytes from obese mice [8, 40]. Previously, it was observed that a balanced translation setup is important for normal spindle and chromosomal morphology during oocyte maturation [41, 42]. We conducted L-homopropargylglycine (HPG) nascent protein translation assays and found that MII-stage HFD + IGF2 oocytes displayed a significant increase in overall protein synthesis compared to HFD oocytes (Fig. 5E and F). The potential role of IGF2 in increasing total protein synthesis in vitro in aged mouse oocytes and rat articular chondrocytes were also observed in different studies [38, 43]. Thus, our data suggest that supplementation of IGF2 to the culture medium can improve mitochondrial function and protein translation in oocytes from obese mice.

### IGF2 improves the ultrastructure of mitochondria of oocytes from obese mice

**Fig. 6:**
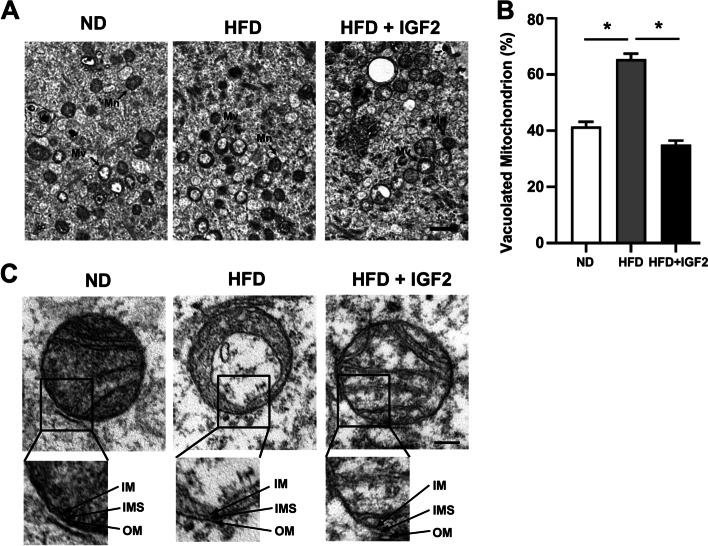
IGF2 improves the mitochondrial ultrastructure of oocytes from obese mice (**A**) Mitochondrial micrograph of ND, HFD, and HFD + IGF2 oocytes at 5000x magnification examined by TEM. Scale bar = 1 μm. Note the normal mitochondria (Mn) and the vacuolated mitochondria (Mv). **B** Numbers of mitochondria per defined region of interest (ROI) in ND, HFD, and HFD + IGF2 oocytes. *p < 0.05. One-way ANOVA test was used for statistical analysis. Error bars indicate the SEM. **C** Mitochondrial micrograph from control and IGF2-treated oocytes at 60,000x magnification. Inner membrane (IM), outer membrane (OM), and intermembrane space (IMS). Scale bar = 100 nm

In light of previous results from a study of aged oocytes, we next examined the functional impact(s) of IGF2 in mitochondrial ultrastructure in oocytes from obese mice. Transmission electron microscopy revealed that HFD + IGF2 oocytes displayed a higher percentage of normal mitochondrial morphology (relative to ND), with well-defined cristae; the HFD oocytes displayed a higher percentage of abnormal morphology (vacuolated cristae) (Fig. 6A and 5B). In addition, HFD + IGF2 oocytes featured prominent inner and outer mitochondrial membranes with proper intermembrane spaces in well-defined form, whereas the HFD oocytes showed disrupted mitochondrial membranes structure and intermembrane spaces which were not visible (Fig. 6C). An in vitro study has demonstrated the potential involvement of IGF2 in improving the mitochondrial structure of muscle cells in the culture medium [44]. Thus, these findings suggest that IGF2 treatment can improve the mitochondrial function of oocytes from obese mice by somehow improving mitochondrial ultrastructure.

### IGF2 reduces the early apoptotic index of obese mice oocytes

**Fig. 7:**
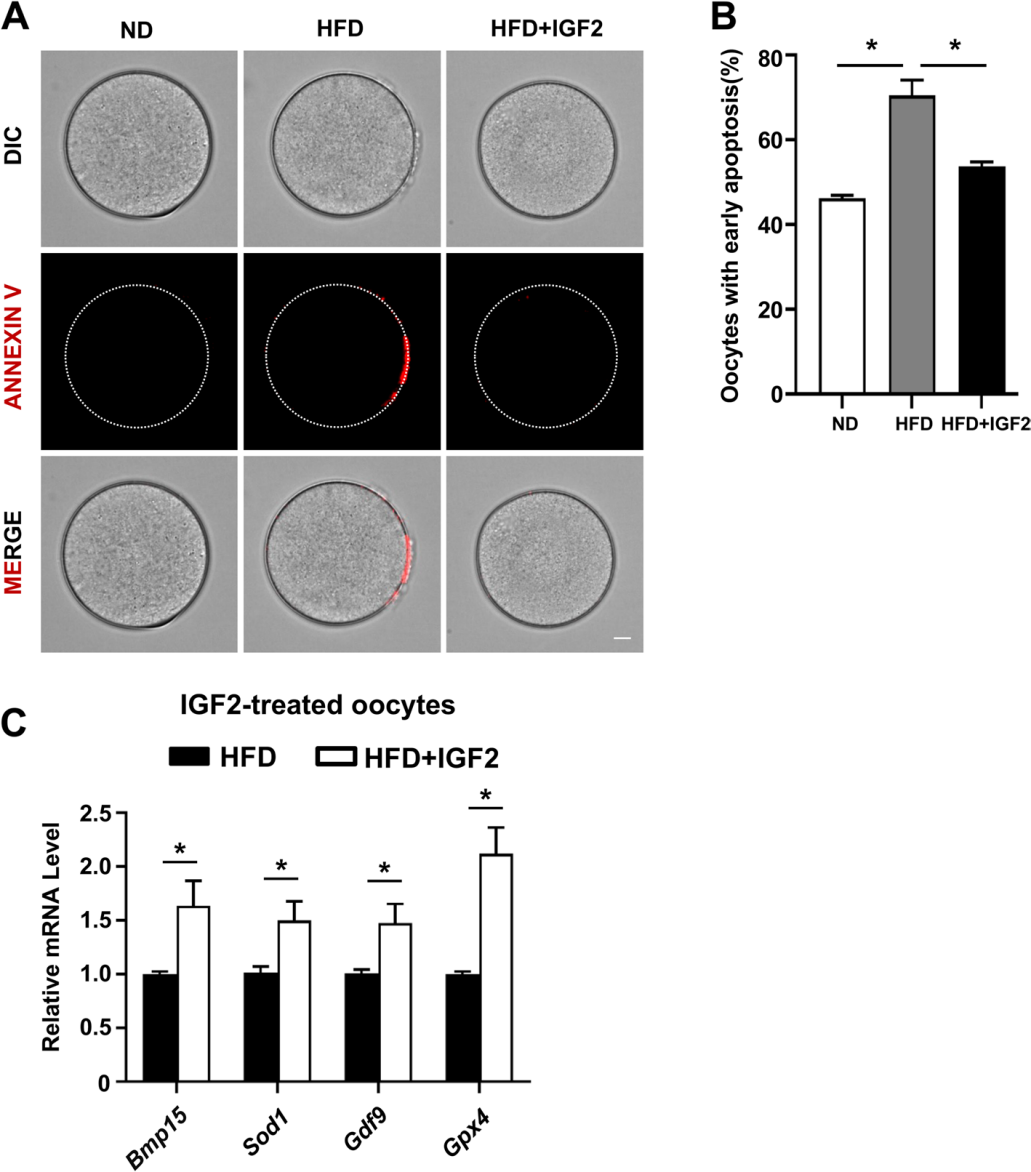
IGF2 reduces apoptosis and induces expression of known target genes in oocytes from obese mice (**A**) Representative images of early apoptosis labeled with Annexin V of ND, HFD, and HFD + IGF2 oocytes. Red fluorescent indicates the Annexin V positive oocytes. Scale bar = 10 μm. **B** The percentage of early apoptosis in MII-stage oocytes from ND (*n* = 100),HFD (*n* = 64) and HFD+ IGF2 (*n* = 62) group. **C** qRT-PCR results showing IGF2 and target genes expression in mouse MII-stage oocytes from ND, HFD, and HFD + IGF2 groups after in vitro maturation. *p < 0.05. One-way ANOVA test and students *t*-test (two-tailed) was used for statistical analysis. Error bars indicate the SEM

Previous studies have reported that impaired mitochondrial functional activity induces oxidative stress and have linked this stress to increased apoptosis in oocytes from obese mice [45, 46]. Moreover, IGF2 reduced apoptotic index was observed in cultured BeWO cells [22]. We, therefore, investigated whether IGF2 administration to the culture medium may impact apoptosis of oocytes from obese mice: Annexin V staining showed that HFD + IGF2 oocytes had a significantly reduced extent of early apoptosis compared to HFD oocytes (Fig. 7A and B). previous reports also have shown the anti-apoptotic signaling mechanism of IGF2 in trophoblast cells of humans and placental cells of mice [22, 47], findings support our data of reduced apoptosis in IGF2 supplemented oocytes from obese mice.

Previous studies have shown that increased mRNA stability of antioxidant genes can predict improved oocytes quality and their development which was previously assessed by the activity of IGF2 in aged mice oocytes [38, 48, 49]. We, therefore, investigated whether IGF2 supplementation to the culture medium may impact these known antioxidant genes expressions of oocytes from obese mice: The HFD + IGF2 oocytes had significantly increased levels of *Bmp15, Sod1, Gdf9*, and *Gpx4* compared to HFD oocytes (Fig. 7C). Thus, IGF2 has the potential to reduce the somehow apoptosis index and can decrease the degradation of mRNAs of genes associated with oocyte development in obese mice.

## Conclusion

Our finding indicates that adding IGF2 to the culture medium can specifically improves the meiotic structures of oocytes from obese mice, ultimately promoting the developmental competency of these oocytes. Our in vivo data confirms reduced IGF2 levels in obese mice and our in vitro work revealed the strong clinical application for deploying IGF2 in fertility clinics for ART purposes to increase the pregnancy outcomes by reducing meiotic defects associated with obesity. Based on previous findings reduced IGF2 contents in infertile female other than obesity was noticed (e.g., oocytes from patients with ovarian hyperstimulation syndrome and polycystic ovarian syndrome), perhaps adding IGF2 to culturing systems may have the potential to improve the revenue of embryos with better quality from these additional populations of women, which may also exert a positive impact on implantation as well as pregnancy rate. Further, investigation based on potential applications of IGF2 for oocytes from obese women, including judging the pregnancy outcome with safety evaluations, is warranted; the feasibility and safety of any IGF2-based clinical interventions still need to be assessed.

## Supplementary Information

**Additional file 1: Sup Fig. 1.** High-fat diet leads to obesity and glucose intolerance in female mice (A,B,C) The body weights of mice receiving HFD for 12 weeks were significantly greater than those of ND mice. (D) Blood glucose monitored in a glucose tolerance test of ND and HFD mice. (E,F) Blood glucose monitored in an insulin tolerance test of ND and HFD mice. *p < 0.05. Student’s *t -*test (two-tailed) was used for statistical analysis. Error bars indicate the SEM.

**Additional file 2: Supplemental Table S1.** Primer sequences for qRT-PCR.

**Additional file 3: Supplemental Table S2.** IGF2 impacts on embryonic developmental competency of oocytes from obese mice.

## Data Availability

The data that support the findings of this study are available from the corresponding author upon reasonable request.
