# Supplementary figures and images for: IGF2 reduces meiotic defects in oocytes from obese mice and improves embryonic developmental competency
Source: Reprod Biol Endocrinol. 2022 Jul 14;20:101. doi: 10.1186/s12958-022-00972-9 (PMC9281013; doi:10.1186/s12958-022-00972-9)

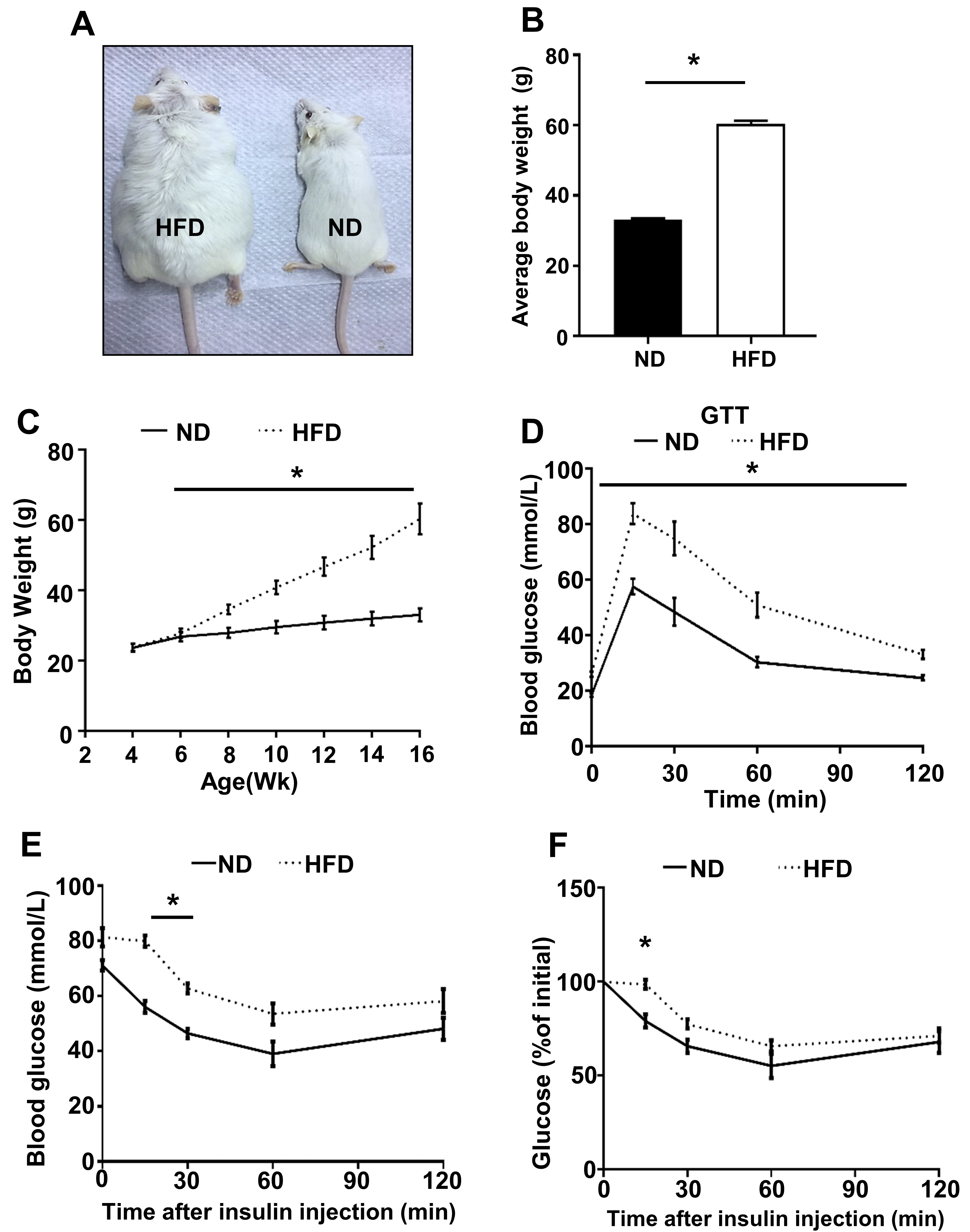

Supplement: Supplementary file 1 — Additional file 1: Sup Fig. 1. High-fat diet leads to obesity and glucose intolerance in female mice (A,B,C) The body weights of mice receiving HFD for 12 weeks were significantly greater than those of ND mice. (D) Blood glucose monitored in a glucose tolerance test of ND and HFD mice. (E,F) Blood glucose monitored in an insulin tolerance test of ND and HFD mice. *p < 0.05. Student’s t -test (two-tailed) was used for statistical analysis. Error bars indicate the SEM. [file 12958_2022_972_MOESM1_ESM.tif]
