# Supplementary material for: IGF2 reduces meiotic defects in oocytes from obese mice and improves embryonic developmental competency
Source: Reprod Biol Endocrinol. 2022 Jul 14;20:101. doi: 10.1186/s12958-022-00972-9 (PMC9281013; doi:10.1186/s12958-022-00972-9)
Supplement: Supplementary file 2 — Additional file 2: Supplemental Table S1. Primer sequences for qRT-PCR. [file 12958_2022_972_MOESM2_ESM.docx]

**Supplemental table 1: Primer sequences for qRT-PCR.**

|  | | **Forward** | **Reverse** |
| --- | --- | --- | --- |
| ***Igf2*** | | AGTCGATGTTGGTGCTTCTCA | CGAACAGACAAACTGAAGCGT |
| ***Bmp15*** | | TCCTTGCTGACGACCCTACAT | TACCTCAGGGGATAGCCTTGG |
| ***Sod1*** | | GCTGTACCAGTGCAGGTCCTCA | CATTTCCACCTTTGCCCAAGTC |
| ***Gdf9*** | TCTTAGTAGCCTTAGCTCTCAGG | | TGTCAGTCCCATCTACAGGCA |
| ***Gpx4*** | ATAAGACGGCTGCGTGGTGAAG | | TAGAGATAGCACGGCAGGTCCTTC |
