# Supplementary material for: IGF2 reduces meiotic defects in oocytes from obese mice and improves embryonic developmental competency
Source: Reprod Biol Endocrinol. 2022 Jul 14;20:101. doi: 10.1186/s12958-022-00972-9 (PMC9281013; doi:10.1186/s12958-022-00972-9)
Supplement: Supplementary file 3 — Additional file 3: Supplemental Table S2. IGF2 impacts on embryonic developmental competency of oocytes from obese mice. [file 12958_2022_972_MOESM3_ESM.docx]

**Supplemental Table S2: IGF2 impacts on embryonic developmental competency of oocytes from obese mice**

|  | | **Morula**  **(%)** | | **Blastocyst**  **(%)** | **Mean cell number/Blastocyst** | |
| --- | --- | --- | --- | --- | --- | --- |
|  | |  |  |  | **TE** | **Total** |
| **ND**  **(n=72)** | 74 | | 61 | | 45.84 | 69.86 |
| **HFD**  **(n=57)** | 60 | | 49 | | 31.40 | 47.60 |
| **HFD+IGF2**  **(n=49)** | 69 | | 61 | | 48.73 | 68.81 |
